# Structural Insights into the Polyphyletic Origins of Glycyl tRNA Synthetases*[Fn FN3]^♦^

**DOI:** 10.1074/jbc.M116.730382

**Published:** 2016-05-23

**Authors:** Marco Igor Valencia-Sánchez, Annia Rodríguez-Hernández, Ruben Ferreira, Hugo Aníbal Santamaría-Suárez, Marcelino Arciniega, Anne-Catherine Dock-Bregeon, Dino Moras, Brice Beinsteiner, Haydyn Mertens, Dmitri Svergun, Luis G. Brieba, Morten Grøtli, Alfredo Torres-Larios

**Affiliations:** From the ‡ Departamento de Bioquímica y Biología Estructural, Instituto de Fisiología Celular, Universidad Nacional Autónoma de México, Circuito Exterior s/n, Ciudad Universitaria, Apartado Postal 70-243, Mexico City 04510, México,; the § Laboratorio Nacional de Genómica para la Biodiversidad, Centro de Investigación y Estudios Avanzados del Instituto Politécnico Nacional, Irapuato, Guanajuato 04510, México,; the ¶ Department of Chemistry and Molecular Biology, University of Gothenburg, SE-412 96 Gothenburg, Sweden,; the ‖ Laboratoire de Biologie Intégrative des Milieux Marins, CNRS UMR8227, 29680 Roscoff, France,; the ** Centre for Integrative Biology, Department of Integrated Structural Biology, Institute of Genetics and of Molecular and Cellular Biology, CNRS UMR 7104, 1 Rue Laurent Fries, Illkirch, France, and; the ‡‡ European Molecular Biology Laboratory, Hamburg Outstation, c/o DESY, Notkestrasse 85, Hamburg 22603, Germany

**Keywords:** aminoacyl tRNA synthetase, crystal structure, molecular evolution, structure-function, substrate specificity

## Abstract

Glycyl tRNA synthetase (GlyRS) provides a unique case among class II aminoacyl tRNA synthetases, with two clearly widespread types of enzymes: a dimeric (α_2_) species present in some bacteria, archaea, and eukaryotes; and a heterotetrameric form (α_2_β_2_) present in most bacteria. Although the differences between both types of GlyRS at the anticodon binding domain level are evident, the extent and implications of the variations in the catalytic domain have not been described, and it is unclear whether the mechanism of amino acid recognition is also dissimilar. Here, we show that the α-subunit of the α_2_β_2_ GlyRS from the bacterium *Aquifex aeolicus* is able to perform the first step of the aminoacylation reaction, which involves the activation of the amino acid with ATP. The crystal structure of the α-subunit in the complex with an analog of glycyl adenylate at 2.8 Å resolution presents a conformational arrangement that properly positions the cognate amino acid. This work shows that glycine is recognized by a subset of different residues in the two types of GlyRS. A structural and sequence analysis of class II catalytic domains shows that bacterial GlyRS is closely related to alanyl tRNA synthetase, which led us to define a new subclassification of these ancient enzymes and to propose an evolutionary path of α_2_β_2_ GlyRS, convergent with α_2_ GlyRS and divergent from AlaRS, thus providing a possible explanation for the puzzling existence of two proteins sharing the same fold and function but not a common ancestor.

## Introduction

Aminoacyl tRNA synthetases are ancient enzymes that attach cognate amino acids to their corresponding tRNAs (1–3). This task is performed in two steps as follows: amino acid activation with ATP, followed by attachment of the resulting aminoacyl adenylate to the cognate tRNA (4–6). According to their sequence and structural features, there are two main classes of non-related aaRS,^4^ which evolved independently from two different domains corresponding to two modes of ATP binding (7–10). These classes are further divided into subclasses according to protein sequence, structural features of the catalytic domain, the presence of accessory domains, and similarity of amino acids (11–13). Most aaRSs are descended from a single ancestor (monophyletic); however, there are two clearly recognized exceptions, lysyl tRNA synthetase and GlyRS (2, 12, 14–16). GlyRS, a class II aaRS, can be found as an α_2_ homodimer in eukarya, archaea, and some bacteria (14, 17–20) and as an α_2_β_2_ heterotetramer in most bacteria and chloroplasts (21–23). Notably, the limited extent of the sequence conservation between the two forms does not allow inference to a common ancestor between them. Nonetheless, the catalytic domains contained in the α-subunits of both GlyRSs share the same fold (11, 14, 16, 22–24). The eukaryotic GlyRS belongs to the subclass IIa, specific for hydrophobic and small polar amino acids, with similarities with HisRS, ThrRS, ProRS, and SerRS. Bacterial GlyRS, however, belongs to the subclass IIc, together with PheRS, AlaRS, SepRS, and PylRS, the most heterogeneous group of the three subclasses (25–27). However, there are some studies that place AlaRS or bacterial GlyRS within subclass IIa and/or do not consider the existence of two types of GlyRS belonging to different subclasses (28–32).

The ∼35-kDa α-subunit of bacterial GlyRS contains the aminoacylation site (22, 24, 33, 34). However, no activity has been demonstrated for this domain alone, suggesting that the β-subunit is required for catalysis (21, 33, 35–37). The ∼65-kDa β-subunit has the most important tRNA recognition elements but is not homologous to the anticodon binding domain of eukaryotic GlyRS (23, 37). Two crystal structures are available for the bacterial GlyRS α-subunit in the apo-conformation (PDB entries 3rf1 and 1j5w), leaving unclear whether the recognition details of the small substrates, glycine and ATP, are shared by the two types of enzymes.

Here, we show that the bacterial GlyRS α-subunit, which has all of the molecular determinants needed for the first step of the reaction, is indeed able to perform this catalysis. The crystal structure of this subunit in complex with an analog of glycyl adenylate (5′-*O*-[*N*-(l-glycyl)sulfamoyl] adenosine, also known as Gly-SA, G5A, or GSAd) allows the conformational changes correlated with glycine recognition to be defined. A comparison of the α-subunit of bacterial GlyRS with the activation domain of archaeal and eukaryotic GlyRS establishes that the two classes of GlyRS employ different chemical strategies to recognize glycine as a substrate. Moreover, a structural and sequence analysis performed on the activation domain of class II aaRS defines a new subclass IId, comprising AlaRS and bacterial α_2_β_2_ GlyRS. This consequently modifies subclass IIc to contain (αβ)_2_ PheRS, SepRS, and possibly PylRS. This classification standing on common structural motifs and active site residues in class IId enzymes allows us to propose a divergence of bacterial GlyRS from AlaRS, which resolves the puzzling existence of two proteins sharing the same fold and function but not a common ancestor.

## Results

### 

#### 

##### α-Subunit of Bacterial GlyRS Is Able to Perform the Amino Acid Activation

**FIGURE 1.**
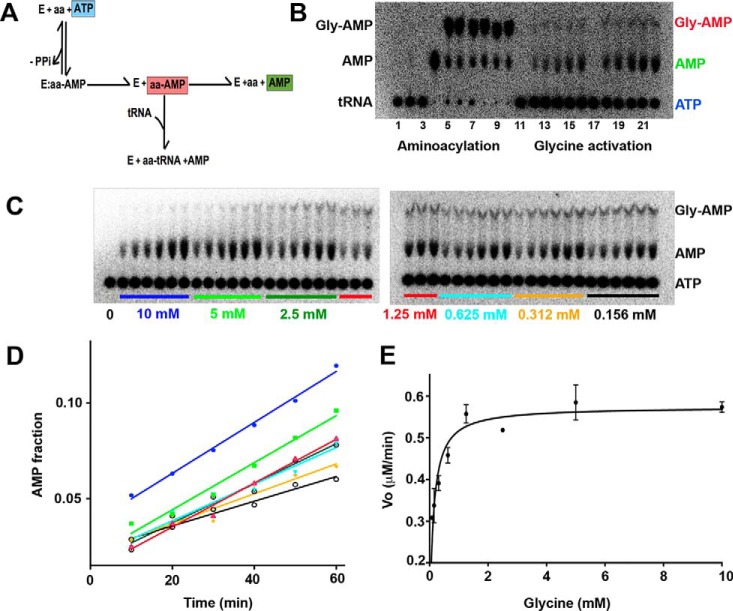
**α-Subunit of the tetrameric α_2_β_2_ GlyRS from *A. aeolicus* (α-AaGlyRS) is able to activate the amino acid.** *A,* schematic diagram of the first step of aminoacyation. *B,* control experiments. Comparison of full aminoacylation reaction *versus* amino acid activation. *Lanes 1–10,* aminoacylation reaction performed as described previously (57–59). *Lane 1*, no enzyme added, and no P1 nuclease added. *Lane 2, Anaeolinea thermophila*, a protein that bears in the same sequence both subunits (αβ-GlyRS) added, and no P1 nuclease added. *Lane 3, A. aeolicus* (α + β-GlyRS) added, and no P1 nuclease added. *Lane 4,* no enzyme added, and P1 nuclease added. *Lanes 5–7,* 5, 10, and 15 min of aminoacylation reaction using α + β-GlyRS (with P1 nuclease added). *Lanes 8–10,* 5, 10, and 15 min of aminoacylation reaction using αβ-GlyRS (with P1 nuclease added). *Lanes 11–22*, glycine activation reaction. *Lane 11*, zero time point using α-AaGlyRS. *Lanes 12–16,* 10, 20, 30, 40, and 50 min of the glycine activation reaction using α-AaGlyRS. *Lane 17*, zero time point using αβ-GlyRS. *Lanes 18–22*, 10, 20, 30, 40, and 50 min of the glycine activation reaction using the αβ-GlyRS. *C,* amino acid activation. α-AaGglyRS at 40 μm, in the presence of decreasing glycine concentrations, 0.5 mm ATP, 50 mm Tris, pH 8.0, 50 mm KCl, 10 mm MgCl_2_. Time points were taken every 10 min for 60 min for each concentration, and the formation of AMP was monitored for each point. *D,* initial velocities (kinetics of AMP formation from the experiment in *C*). Steady state time courses using different glycine concentrations. *E,* Michaelis-Menten plot. Initial velocities were plotted against substrate concentration; *error bars* indicate the standard deviation for each point.

We were able to obtain highly purified α-subunit GlyRS of the hyperthermophilic bacterium *Aquifex aeolicus* (α-AaGlyRS) by means of a heat treatment and an astringent His tag affinity chromatography step. A final purification step using size exclusion chromatography coupled to multiangle light scattering (SEC-MALS) indicated a homogeneous dimeric population of 69.1 kDa (theoretical mass = 67.4 kDa), in agreement with previous reports (21, 36). Small angle x-ray scattering (SAXS) further confirmed the dimeric nature of the ensemble. Because of the evolutionary conservation of all amino acids involved in glycine activation (see below), we speculated that the α-subunit alone would be able to catalyze the first step of the reaction, the attachment of glycine to ATP. With the use of an alternative method based on thin layer chromatography to monitor the activity, we found that α-AaGlyRS was indeed able to perform the first step of aminoacylation (Fig. 1). In contradiction with previous reports, the α-subunit showed weak activity at pH values ranging from 6.0 to 8.0 and glycine concentrations from 80 μm to 10 mm. Under the best possible reaction conditions, the observed *K_m_* for glycine was 0.11 ± 0.016 mm, similar to a previously reported value for the full-length *Escherichia coli* enzyme (21). Nevertheless, the *k*_cat_ reaction was extremely slow, only 2.3 × 10^−4^ s^−1^. Previous studies have performed an ATP-PP_i_ exchange assay, which follows the reverse reaction of incorporation of ^32^P from PP_i_ into ATP (21, 33, 35–37), whereas in our study the Gly-AMP synthesis was measured directly. These findings provide biological relevance to our reported crystal structure (see below) and support the functional existence of an α_2_β_2_ quaternary structure, rather than an (αβ)_2_ organization.

##### Binding to a Transition State Analog Promotes Conformational Changes in the α-Subunit of Bacterial GlyRS

**FIGURE 2.**
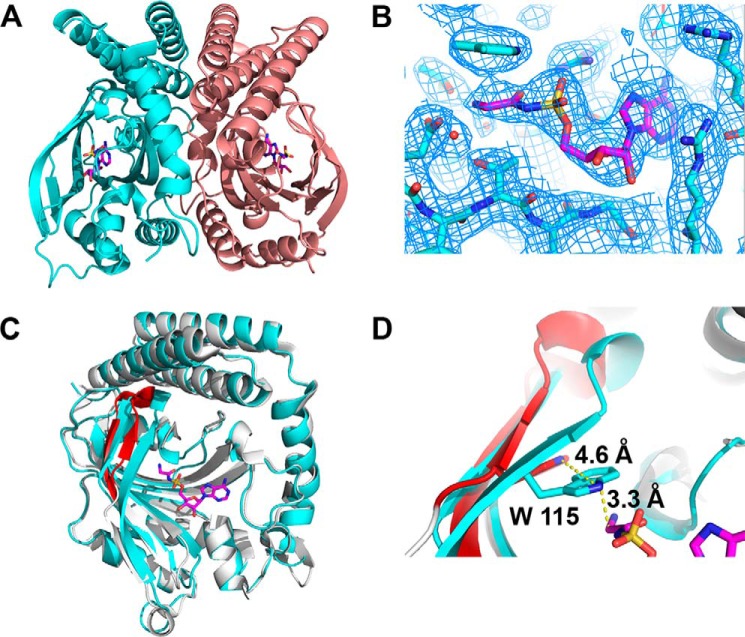
**Binding of a glycyl adenylate analog promotes a conformational change in α-AaGlyRS.** *A,* overall dimeric structure of α-AaGlyRS with GSAd shown in *magenta*. There are five monomers, each with a ligand bound, in the asymmetric unit. *B,* simulated annealed, 2*F_o_* − *F_c_*, omit electron density map contoured at 1σ around the GSAd-binding site. Two solvent molecules were located near the glycine recognition site that could be well fitted and observed on double difference and omit maps for monomer B and could be seen as peaks bigger than 3.5σ on difference, *F_o_* − *F_c_* electron density maps on the four other monomers. No other solvent molecules were added to the model. *C,* superposition of α-AaGlyRS-GSAd with the apo structure of *Campylobacter jejuni* (PDB code 3rgl). The sequences of the subunits have an identity of 60% and a similarity of 77%. The overall r.m.s.d. is 0.959 Å. The binding of GSAd causes conformational changes in the region of residues 112–123, shown in *red* in *C* and *D. D,* close-up of the superposition showing the conformational change upon binding of GSAd, which involves a 4.6 Å movement of Trp-115 to make a cation-π interaction with GSAd. The superposition with another α-subunit structure from *T. maritima* (PDB code 1j5w) does not show this displacement, suggesting that this conformational change is due to GSAd binding.

To understand amino acid and nucleotide recognition in bacterial GlyRS, we solved the crystal structure of α-AaGlyRS in complex with GSAd at 2.81 Å resolution (Table 1). The electron density map unambiguously showed all features of the bound GSAd and its molecular surroundings in all five molecules in the asymmetric unit (Fig. 2*B*).

**TABLE 1:** **Data collection and refinement statistics** One crystal was used.

	α-AaGlyRS-GSAd
**Data collection**	
Space group	P22_1_2_1_
Cell dimensions	
*a*, *b*, *c* (Å)	101.8, 130.0, 145.5
α, β, γ (°)	90.0, 90.0, 90.0
Resolution (Å)	83.43–2.81 (2.91–2.81)*^a^*
*R*_sym_ or *R*_merge_	0.084 (0.584)
*I*/σ*I*	9.7 (2.2)
Completeness (%)	100 (100)
Redundancy	4.4 (4.5)
Mean (*I*) half-set correlation *CC*_1/2_	0.990 (0.770)
Wilson *B*-factor (Å^2^)	58.4

**Refinement**	
Resolution (Å)	80.19–2.81 (2.86–2.81)
No. of reflections	47,741(2607)
*R*_work_/*R*_free_	0.239/0.252 (0.362/0.363)
No. of atoms	
Protein	11,740
Ligand/ion	135
Water	2
*B*-Factors	
Protein (by chain)	45.1, 53.6, 56.9, 69.6, 73.1
Ligand/ion	40.8, 47.5, 52.6, 68.0, 74.6
Water	45.8
r.m.s.d.	
Bond lengths (Å)	0.011
Bond angles (°)	1.462

*^a^* Highest resolution shell is shown in parentheses.

As shown previously (PDB code 1j5w (24)), the α-subunit forms a homodimer (Fig. 2*A*), with 58% of its 2492 Å^2^ interface formed by a 97-residue helical region located at the C-terminal part of the subunit and situated on top of the signature antiparallel β-strand of class II synthetases. Further details on the description of the general architecture of this subunit have been given previously (24).

**FIGURE 3.**
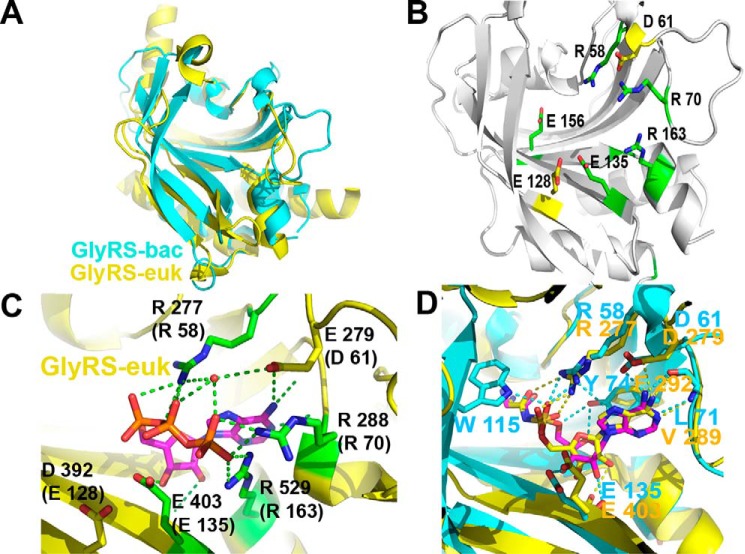
**Features shared by the two types of GlyRSs.** *A,* superposition of the catalytic core, according to Dali, of bacterial GlyRS (residues 1–169) and eukaryotic GlyRS (residues 97–536, with the exception of extensions comprising residues 129–137, 145–242, and 301–349, PDB code 2zt8). The Z-score is 14.9 for the 152 residues aligned, with an r.m.s.d. of 2.7 and 17% identity. *B,* residues that are highly (*yellow*) or absolutely conserved (*green*) and also shared by bacterial GlyRS and eukaryotic GlyRS according to the structural and sequence alignment of all available sequences are shown on the structure of bacterial GlyRS. The following panels show the role and/or location of some of these residues in more detail. *C,* five of the seven residues depicted in *B* are highly conserved class II residues that recognize ATP (PDB code 2zt7). *D,* in comparison with eukaryotic GlyRS, α-AaGlyRS Tyr-74 makes additional interactions through its OH group, which contacts the O4′ and O5′ atoms of GSAd and the NE1 atom of Trp-115 that contacts the O1S atom of GSAd.

Comparison of α-AaGlyRS with previously solved structures of this subunit in the apo-form reveals conformational changes in the region formed by residues 112–123 (Fig. 2, *C* and *D*). This segment is the topological equivalent of the so-called amino acid loop found in HisRS, ProRS, ThrRS, SerRS, and eukaryotic GlyRS (38). Here, the movement of this region causes an ∼5 Å displacement of Trp-115 when compared with the apo structure, allowing it to form a cation-π interaction with the glycine moiety of GSAd (Fig. 2*D*, see below) and to form a hydrogen bond with the O1S atom of GSAd (equivalent to the O1P atom of the cognate Gly-AMP (Fig. 3*D*)). As a result, the size of the active site pocket is reduced from an area and volume of 706 Å^2^ and 960 Å^3^ to 451 Å^2^ and 636 Å^3^.

In correlation with this movement, thermal shift assays showed a displacement of the *T_m_* values of α-AaGlyRS in the presence of GSAd, from 83.2 °C (no ligand) to 92.3 °C (8 mm GSAd). We could not detect significant changes in the presence of glycine, ATP, or ATP + glycine. The reported crystal structure of the α-subunit of bacterial GlyRS shows a productive, biologically meaningful complex that allows the recognition mechanisms in the two types of GlyRS to be compared.

##### Mechanism of Glycine Recognition Differs in the Two Types of Known GlyRS

**FIGURE 4.**
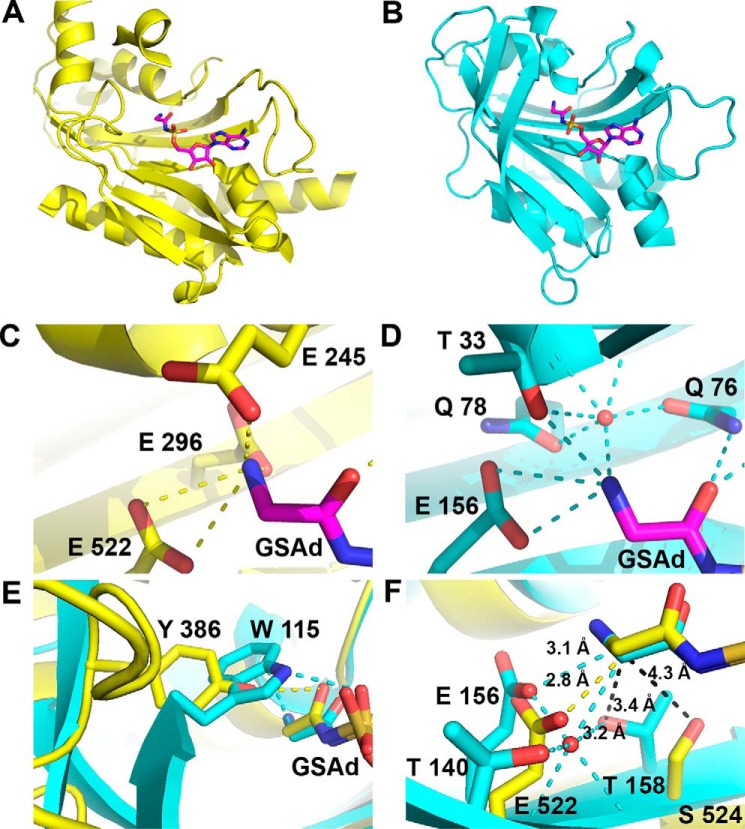
**Distinct modes of amino acid recognition in polyphyletic GlyRSs.** *A, ribbon* diagram of the catalytic core of eukaryotic GlyRS (PDB code 2zt8). *B, ribbon* diagram of the catalytic core of bacterial GlyRS. *C, stick* diagram of residues involved in the amino group of glycine recognition in eukaryotic GlyRS. *D, stick* diagram of residues involved in the amino group of glycine recognition in bacterial GlyRS. Thr-33 and Gln-76 have no topological equivalent in eukaryal GlyRS. All residues involved in the amino group recognition are absolutely conserved in each type of GlyRS. *E,* non-conserved Tyr-386 of the amino acid loop in eukaryotic GlyRS (*yellow*) compared with the fully conserved Trp-115 of bacterial GlyRS (*cyan*). *F,* discrimination of non-cognate amino acids by steric hindrance. Non-conserved Ser-524 in eukaryotic GlyRS (*yellow*) compared with highly conserved Thr-158 and 140 in bacterial GlyRS (*cyan*).

Although nucleotide recognition shows common features in both types of GlyRSs (Fig. 3), critical differences are found at the amino acid recognition level, which is centered on the amino group. In eukaryotic GlyRS, the amino group is recognized by three conserved and negatively charged glutamic acid residues (Glu-522, Glu-296, and Glu-245 in PDB code 2zt8) (Fig. 4, *A* and *C*) (39). In contrast, in the bacterial type GlyRS, the amino group is recognized through five conserved different residues (Trp-115, Gln-76, Gln-78, Thr-33, and Glu-156) (Figs. 2*D* and 4, *B, D,* and *E*). Interestingly, Gln-76 interacts directly with the glycine carbonyl group and also with the amino moiety through hydrogen bonding, with Gln-78, of a solvent molecule (Fig. 4*D*). The amino group of GSAd also interacts with the side chains of Thr-33 and Glu-156 (Fig. 4, *B* and *D*).

The differences with eukaryotic GlyRS are also extended to the region of the amino acid loop. In eukaryotic GlyRS, the hydroxyl group of a non-conserved Tyr-386 contacts the carbonyl group of GSAd (Fig. 4*E*). In contrast, in bacterial GlyRS, an absolutely conserved Trp-115 makes a clear cation-π interaction with the amino moiety of GSAd and also contacts the O1S atom of GSAd (Figs. 2*D* and 4*E*).

In addition, in eukaryotic GlyRS, the binding of the noncognate alanine is prevented in part by a non-conserved Ser (or Ala) residue (524) and the highly negatively charged recognition cavity (Fig. 4, *C* and *F*). In contrast, in bacterial GlyRS, this steric hindrance is made by a pair of highly conserved Thr residues (158 and 140) together with a nearby solvent molecule (Fig. 4*F*).

The structural sequence alignment combined with multiple sequence alignments indicate seven highly conserved residues that are shared by the two types of GlyRS (Fig. 3*B*). Five of these are involved in ATP recognition, and as such, these residues are highly conserved and shared by many aaRSs. There is only one residue that is shared by the two types of GlyRS for the recognition of glycine (Glu-156 in bacterial GlyRS and Glu-522 in eukaryotic GlyRS). Not another single residue is shared in any other region of the catalytic domain. Comparison of the electrostatic potentials within the glycine binding pockets shows a highly negatively charged region in eukaryotic GlyRS and a less polar environment in bacterial GlyRS. The comparison of the two types of GlyRSs and further analysis as shown below indicate that their differences are so profound, up to the level of amino acid recognition, that it is extremely unlikely for them to share a common ancestor, even when their catalytic domains have the same overall fold.

##### Bacterial GlyRS Presents Key Differences Compared with Other Class II Synthetases

**FIGURE 5.**
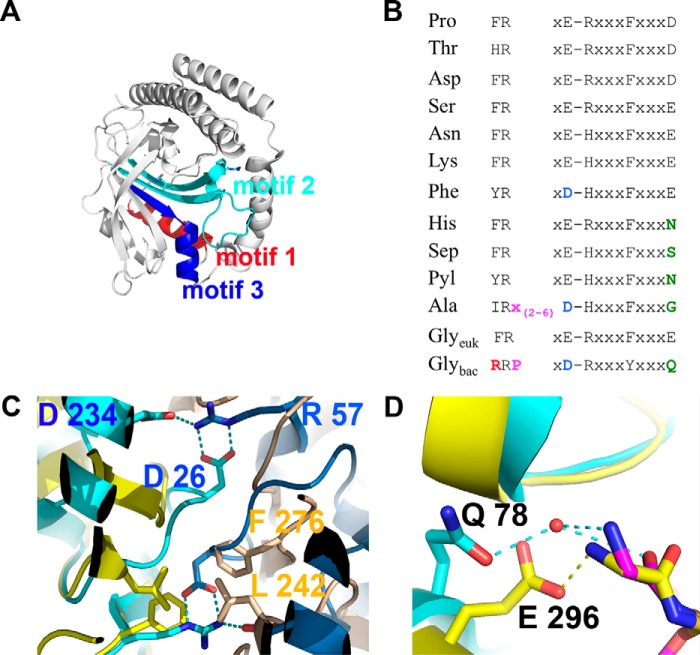
**Bacterial GlyRS shows key differences with class II aaRSs.** *A,* three class II motifs are color-coded and labeled on the bacterial α-GlyRS monomer. Motif 1, residues 3–17; motif 2, residues 48–82; and motif 3, residues 155–169. *B,* signature sequences of motif 2. The consensus sequences were obtained from the analysis of the seed alignments from the Pfam families PF00587 (subclass IIa), PF00152 (subclass IIb), PF01409 (PheRS), PF01411 (AlaRS), and PF02091 (bacterial GlyRS), as well as from our sequence analysis based on structure using Multiseq (see “Experimental Procedures”). The four main differences of bacterial GlyRS with most of class II synthetases are marked in color. Arg-57 and Gln-78 are further analyzed in *C* and *D*, respectively. *C,* differences at the dimerization interface (Arg-57). In bacterial GlyRS (*cyan* and *blue*), Arg-57 mediates a salt bridge with Asp-26 and Asp-234. In eukaryotic GlyRS (*yellow* and *light orange*), a hydrophobic patch made by Phe-276 and Leu-242 is found in the equivalent region. In some sequences, an Ile residue substitutes Arg-57. This change is correlated with a substitution of Asp-26 by a Thr or Asn residue. *D,* differences in the cognate amino acid binding pocket. Gln-78 in bacterial GlyRS (*cyan*) helps to bind a solvent molecule that recognizes in part the amino group of the cognate amino acid. In the equivalent position in all class II aaRS, exemplified with eukaryotic GlyRS (*yellow*), Glu-296 helps to recognize the amino group of glycine.

A hallmark of class II synthetases is the presence of key residues located in each of their three characteristic motifs (Fig. 5*A*) (7). In bacterial GlyRS, the signature sequence of motif 2, formed by the first two strands of the antiparallel β-sheet that forms the floor of the active site, presents several changes compared with the consensus sequence (Fig. 5*B*). In particular, there are three key differences. 1) Unique to bacterial GlyRS, an arginine residue (Argo57) is located where a hydrophobic residue is otherwise always present (Fig. 5, *A–C*). Additionally, an Arg residue makes a salt bridge with two highly conserved Asp residues (Fig. 5*C*). In the rest of class II synthetases, a Phe, Tyr, Val, or His residue helps to stabilize a hydrophobic region located at the dimeric interface (Fig. 5*C*). 2) Similar to AlaRS, there is an insertion (Pro-59 in bacterial GlyRS and two to six residues in AlaRS) between an absolutely conserved Arg and an acidic residue (Fig. 5, *A* and *B*). 3) In many aaRSs, a fully conserved acidic residue recognizes the amino moiety of the cognate amino acid (Fig. 5, *A*, *B* and *D*). In bacterial GlyRS, AlaRS, HisRS, SepRS, and PylRS, another residue occupies this position (Fig. 5*B*). In bacterial GlyRS, a glutamine residue is substituted in place of this acidic residue (Fig. 5, *A*, *B* and *D*). In α-AaGlyRS, Gln-78 makes a hydrogen bond with a solvent molecule that interacts with the amino moiety of the cognate glycine (Fig. 5*D*). These sequence-structure observations confirm that bacterial GlyRS has non-canonical features that are somewhat shared with other atypical aaRSs, like AlaRS, and not with eukaryal GlyRS.

##### Bacterial GlyRS and AlaRS Form a Structural Subclass of aaRS

**FIGURE 6.**
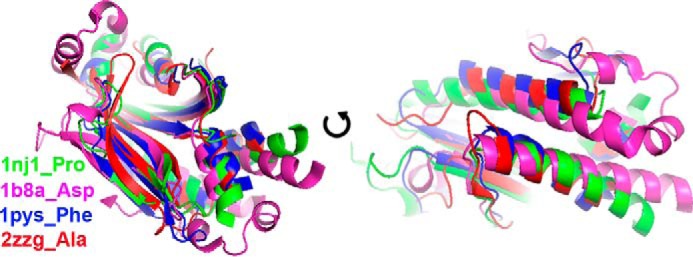
**Initial selection of a catalytic core of class II aaRSs.** A collection of 111 crystal structures from class II aaRSs was chosen according to their diversity in specificity, species, and ligands bound in the active site. The catalytic cores of these structures was selected by including the residues located between motifs 1 and 3 and excluding additional insertions or domains not including common structural motifs for most aaRSs. An overall multiple structural alignment was made using the STAMP algorithm (60) as implemented in MultiSeq in VMD (61, 62). This panel shows the superposition of structures of the catalytic core described and used to obtain the Dali Z-score matrix shown in Table 2, as well as the dendrograms shown in supplemental Fig. S1 (based on structure according to STAMP) and supplemental Fig. S2 (based on sequence according to T-Coffee and built with PhyML). It represents a structure from each obtained subgroup.

**FIGURE 7.**
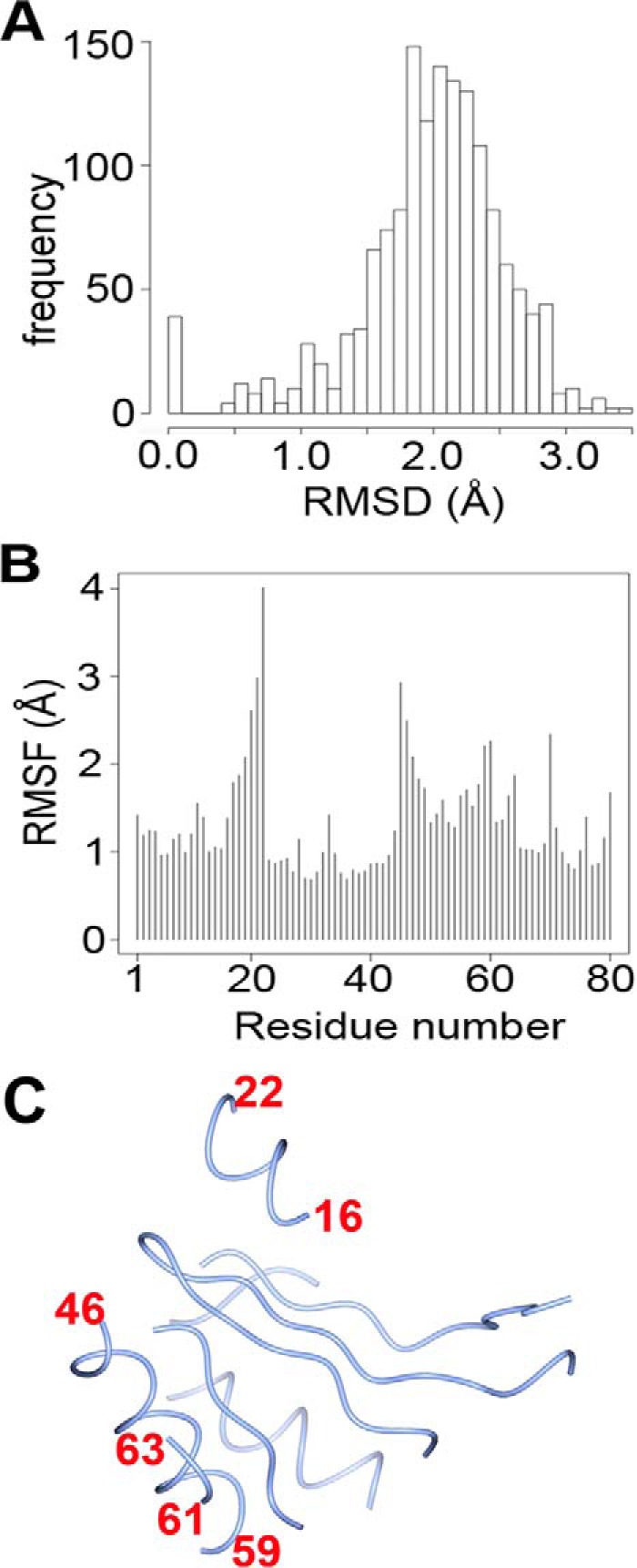
**Structural features of the conserved core of 80 Cα atoms of class II aaRSs.** 39 structures from the alignment described in Fig. 6 were further trimmed, and the largest positional differences were excluded to determine the invariant core of class II aaRSs using the Bio3D package in R (68). An r.m.s.d. and a PCA, shown in Figs. 8 and 9, were performed using the 80 residues core depicted in *C. A,* histogram of pairwise r.m.s.d. values *B,* root mean square fluctuations (*RMSF*) plot. *C,* location on the 80-residue core of the main differences shown in *B*.

**FIGURE 8.**
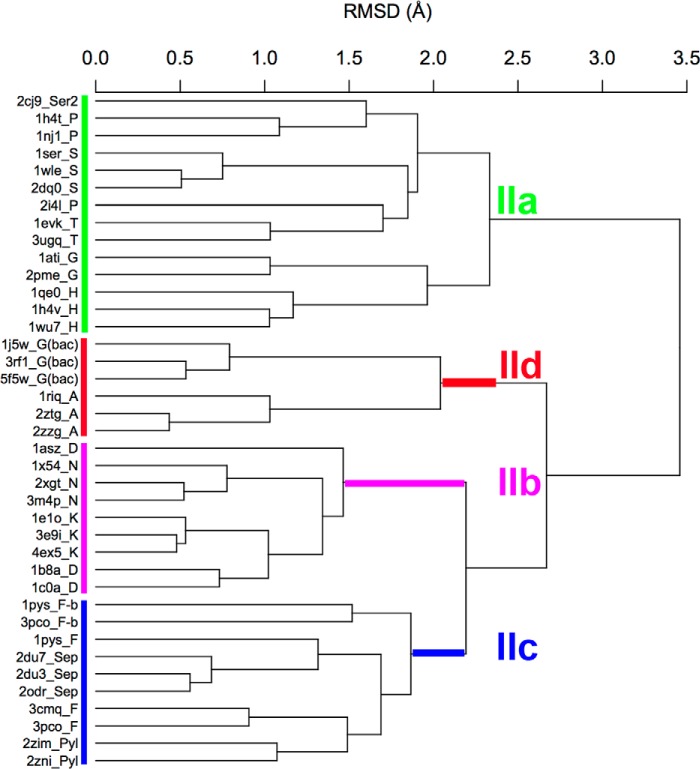
**Bacterial GlyRS and AlaRS form a subclass of aaRSs.** r.m.s.d. cluster dendrogram of classII aaRSs. The dendrogram was calculated according to the Cα core of 80 residues (see Fig. 7). This tree is in full agreement with previous subclass definitions but with an additional subgroup formed by bacterial GlyRS and AlaRS.

**FIGURE 9.**
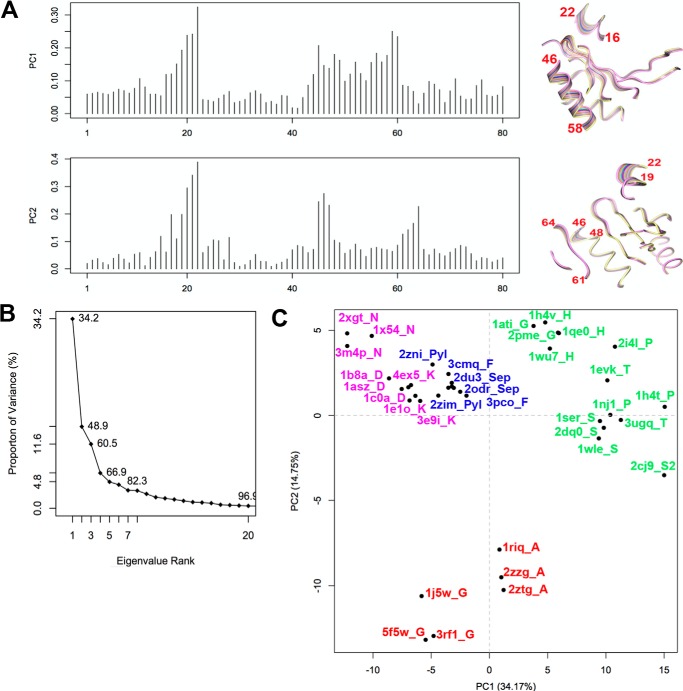
**Bacterial GlyRS and AlaRS form a subclass of aaRSs.** PCA of classII aaRSs. *A,* contribution of each of the 80 residues Cα core, as defined in the analysis described in Fig. 7, to the first two principal components found. *B,* contribution of each component to the total variance of the distribution of structures. *C,* plot along PC1 and PC2, which describe the change that covers 49% of the structural variance in class II aaRSs.

**FIGURE 10.**
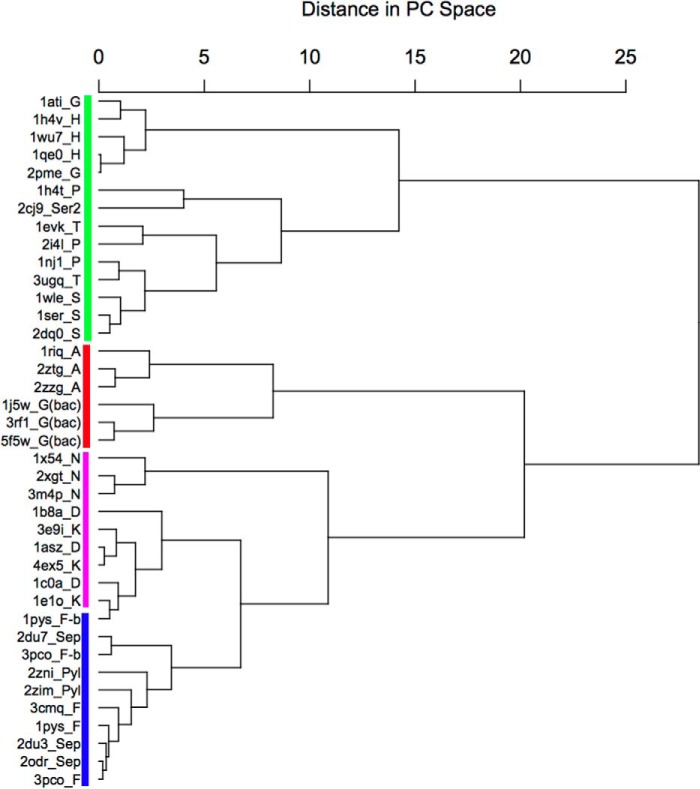
**PCA cluster dendrogram of classII aaRSs.** Clustering dendrogram along PC1 and PC2 as described in Fig. 9.

**FIGURE 11.**
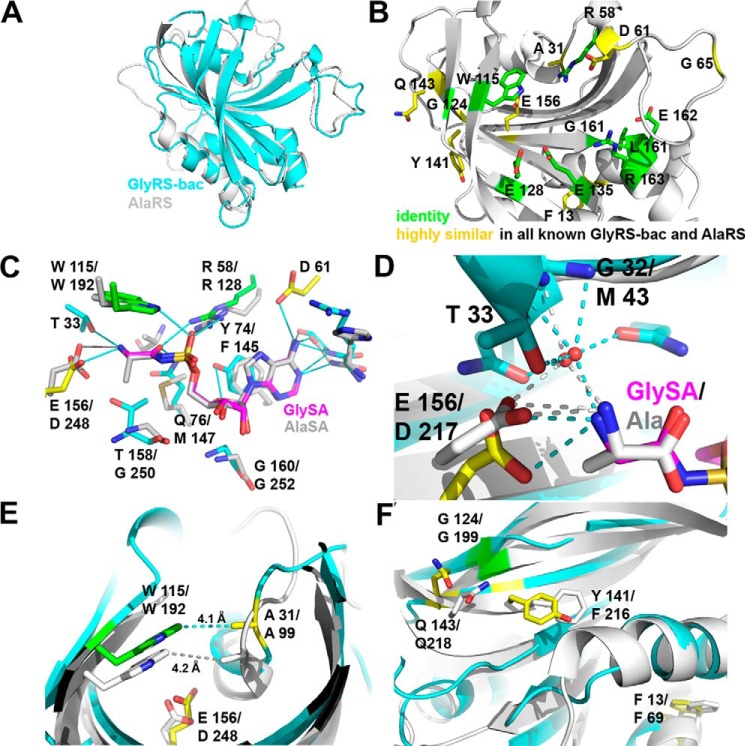
**Bacterial GlyRS and AlaRS share several features.** *A,* superposition of the catalytic core, according to Dali, of bacterial GlyRS (residues 1–169) and AlaRS (PDB code 2zzg, residues 58–260). The Z-score is 19.5 for the 160 residues aligned, with r.m.s.d. of 2.3 and 22% identity. *B,* residues that are highly (*yellow*) or absolutely conserved (*green*) and also shared by bacterial GlyRS and AlaRS, according to the structural and sequence alignment of all available sequences, are shown on the structure of bacterial GlyRS. The following panels show the role and/or location of some of these residues in more detail. *C,* superposition of the active site region. Some shared residues (Trp-115/Trp-192) and Glu-156/Asp- 248 are related with the cognate amino acid recognition. Thr-158/Gly-250 are proposed to be related with amino acid discrimination. Some other residues (Arg-58/Arg-128), for example, are common class II residues aimed to recognize the ATP molecule. *D,* amino group of the cognate amino acid is in part recognized by a solvent molecule and an acidic residue (Glu-156/Asp-217) in both bacterial GlyRS and AlaRS (PDB code 1yfs, in complex with Ala). A positive electron density peak is also seen in the same position of the solvent molecule for PDB code 2zzg, the model that was used for all the figures of this enzyme shown in this work. *E,* Trp-115/Trp-192 makes a cation-π interaction on the cognate amino acid that helps to close the active site cavity, together with Ala-31/Ala-99 located in the vicinity. *F,* four other residues are absolutely or highly conserved in both enzymes and are not related to substrate recognition.

To gain further insight into the evolutionary origin of bacterial GlyRS, we performed pairwise structural alignments of the catalytic core (proposed to reflect the primordial synthetase (40)), among the available crystal structures of all types of class II aaRS using two different algorithms (Dali (41) and STAMP (42)). We also performed a multiple sequence alignment using the structural information through T-Coffee Expresso (43). Finally, we performed a principal component analysis (PCA) on the Cα atoms of 80 core residues common to the structure of class II aaRSs, according to the algorithm implemented in Bio3D (44). The information derived from each of the four approaches is summarized in Figs. 6–10, Tables 2 and 3, and supplemental Figs. S1 and S2. We were able to clearly identify four subclasses of class II aaRSs. These subclasses could be defined either using the whole catalytic domain just devoid of big insertions (Fig. 6, Table 2, and supplemental Figs. S1 and S2) or a small Cα core of 80 atoms (Figs. 7–10). The analysis of the Cα core of 80 atoms showed that the main differences between the groups are found in two helical regions and one β-strand (PC1 and PC2, residues 16–22, 46–58, and 61–64 of the Cα core, Figs. 7 and 9*A*). Notably, the PCA analysis (Fig. 9) shows that only these two components, which together account for almost 50% of the structural variance of the core between the class II structures (Fig. 9*B*), allow us to define a clear separation between the subgroups, most importantly, the group formed by bacterial GlyRS and AlaRS and the rest of class II aaRSs (Figs. 9*C* and 10). The four subgroups are maintained even if bacterial GlyRS or AlaRS is removed from the PCA. At the whole catalytic domain level, the superposition of representatives of the four subclasses shows a general fold agreement (Fig. 6, average Dali Z-score of 13.4). A closer approach allows us to visualize the differences among the four established divisions (groups with Dali Z-scores equal to or higher than 18.5, which turned out to be equal to the branches of the STAMP analysis based on r.m.s.d.) and the agreement between them (Fig. 6, Table 2, and supplemental Fig. S1). Each subclass is mostly defined by the relative angles of two helices located at the back of the active site (subclass IIa *versus* IIb), by the length of these helices (subclass IIa and IIb *versus* IIc and IId), or by the length and relative orientation of three strands of the active site β-sheet (subclass IIc *versus* IId). Notably, each subclass is in full agreement with previously proposed divisions. However, subclass IIc is now divided in two distinct subclasses: IIc, including PheRS, SepRS, and possibly PylRS, which groups with subclass IIc according to the structural classification (Figs. 8–10 and supplemental Fig. S1) but with subclass IIb according to the sequence analysis (supplemental Fig. S2). The other subclass, IId, includes AlaRS and bacterial GlyRS, which match not only in helical length but also the length and orientation of three strands of the active site β-sheet (Fig. 11).

**TABLE 2:**
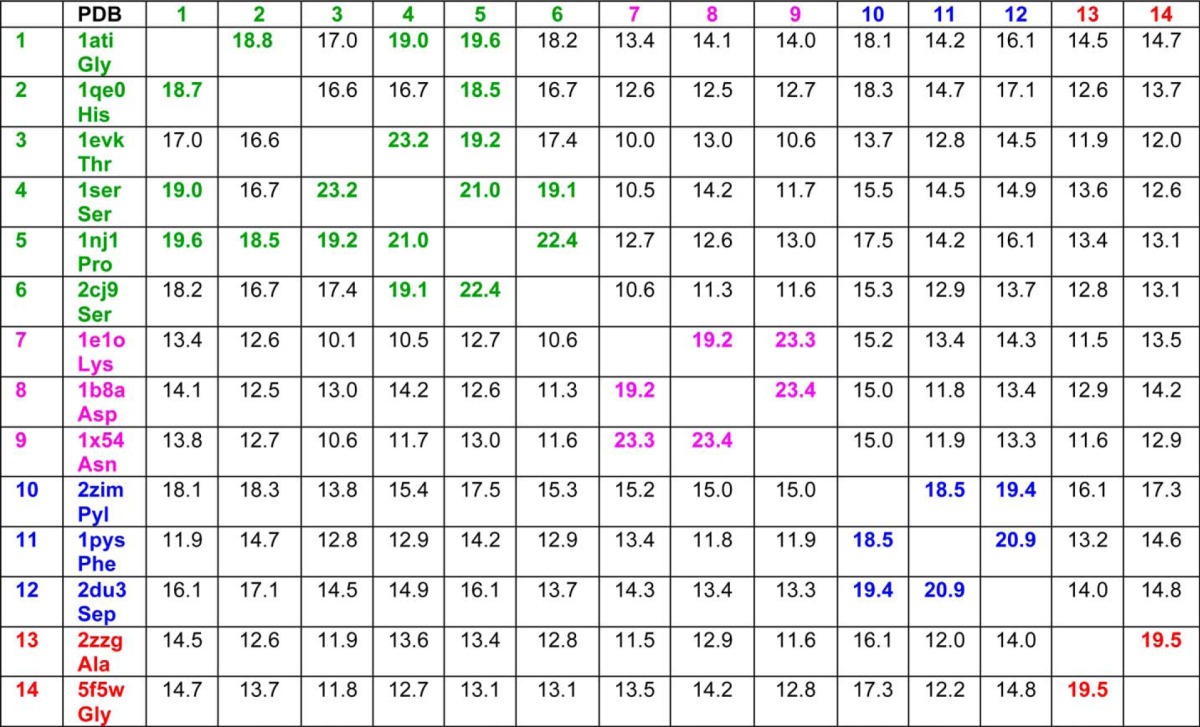
**DALI Z-score of pairwise comparisons of representative structures of class II aaRSs** Z-scores higher than 18.5 are indicated in bold and grouped by colors that indicate class II subclasses: a, (green); b, (magenta); c, (blue); d, (red).

**TABLE 3:**
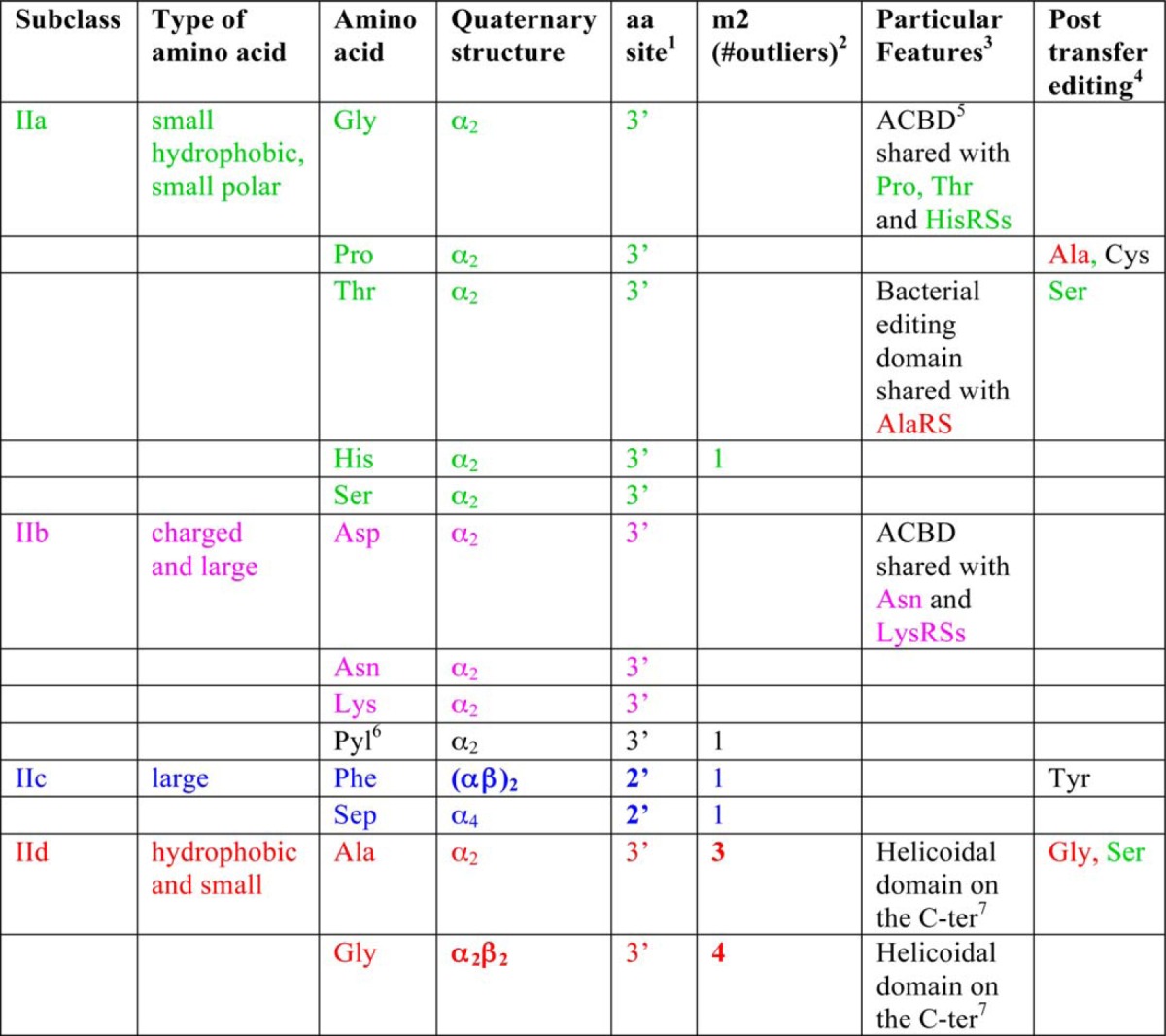
**Proposed subgroups of class II aaRSs**

^1^ Aminoacylation site (2′ or 3′ OH of the terminal ribose of the cognate tRNA).

^2^ Number of residues in motif two that do not agree with the canonical sequence.

^3^ Although this proposed classification is based solely on the structural and sequence features of the catalytic core of class IIaaRSs, there are some characteristics that are shared among its members.

^4^ Editing carried out after the first step of the reaction against standard amino acids.

^5^ ABCD is anticodon binding domain.

^6^ PylRS can be either subclass IIc according to the structural analysis or subclass IIb according to the sequence analysis. It is placed in the frontier in this table because of the aminoacylation site properties of PheRS and SepRS, which are different from PylRS.

^7^ These domains have limited similarity between each other (Dali Z-score 2.6, r.m.s.d. 4.5 Å, % identity = 12).

##### Bacterial GlyRS and AlaRS Share Key Residues Important for Both Protein Structure and Substrate Recognition

The structural sequence alignment coupled with multiple sequence alignments (Fig. 11, *A* and *B*) indicate that there are 15 residues either absolutely or highly conserved by bacterial GlyRS and AlaRS. Five of these are involved in ATP recognition (Fig. 11, *B* and *C*). Outside of this, the amino acid recognition region surprisingly revealed that the equivalent of Trp-115 in bacterial GlyRS is also found in AlaRS (Trp-192, PDB code 2zzg) (Fig. 11, *B, C* and *E*) (45). This residue is absolutely conserved in AlaRSs from all domains of life and bacterial GlyRS and only in these synthetases. Notably, there is a conformational change in the region of Trp-192 in AlaRS in response to ligand binding (45), similar to the one seen in bacterial GlyRS. There is also a highly conserved Ala-31 (Ala-99 in AlaRS), which is 4 Å from Trp-115 (Fig. 11, *B* and *E*), besides an equivalent of Glu-156 (Asp-248 in AlaRS) that recognizes the amino group of the cognate amino acid (Fig. 11, *B*, *C* and *D*). In addition, the location of a water molecule that interacts with the amino moiety of the cognate amino acid (Fig. 11*D*) complements the remarkable similarities in the active site cavity. Interestingly, a partial solvent-mediated recognition of the amino group is also found, for example, in ThrRS (46).

Furthermore, there are four residues, outside the active site cavity, that are highly conserved in AlaRS and bacterial GlyRS (Fig. 11, *B* and *F*). These amino acids are Phe-13, Gly-124, Tyr-141, and Gln-143 in bacterial GlyRS, which correspond to Phe-69, Gly-199, Phe-216, and Gln-218 in AlaRS (Fig. 11, *B* and *F*). As mentioned previously, there are no such residues, indicative of a much higher sequence-structure relatedness, in the comparison of both types of GlyRS.

Taken together, the structural and sequence similarities between the catalytic domains of AlaRS and bacterial GlyRS demonstrate a greater degree of conservation than that observed between the two types of GlyRSs. These similarities and differences allow for a new subclass of aaRSs to be defined that is clearly different from any other subgroup.

## Discussion

In this work, we explored three long-standing questions regarding bacterial GlyRS. The first was based on previous experimental evidence suggesting that the full α_2_β_2_ tetramer was needed for any reaction step. The second concerned the comparison of substrate recognition and the active sites of the two types of GlyRSs at the molecular level. Finally, the third question deals with the subclassification of class IIc aaRSs and the possible origin of bacterial GlyRS.

### 

#### 

##### α-Subunit of Bacterial GlyRS Is Dimeric and Active for the First Step of Aminoacylation

The α-subunit of bacterial GlyRS contains all the determinants for aminoacyl-adenylate synthesis, and here we show that the activation of glycine can indeed take place with this chain alone. Given the high sequence conservation of this subunit in bacteria (Pfam family PF2091), it is likely that the same observation would apply to all available enzymes.

The rigorous purification of the α-subunit, which included a heating step to 75 °C for 30 min (*T_m_* of the α-subunit = 83.2 °C, according to thermal shift assays), nickel-nitrilotriacetic acid affinity purification in the presence of relatively high amounts of salts (1 m KCl and 0.5 m urea), and SEC-MALS purification, allowed us to obtain a homogeneous dimeric population of 69.1-kDa protein, free of even trace amounts of higher molecular weight complexes. Previous works have reported that the α-subunit of bacterial GlyRS contains the aminoacyl adenylate synthesis site, but, unlike this study, no activity had been seen. These findings also support the functional existence of an α_2_β_2_ quaternary structure for bacterial GlyRS, rather than an (αβ)_2_ organization, as seen in most PheRSs (47).

##### Two Types of GlyRS Recognize Glycine in Distinct Ways

The crystal structure of a bacterial GlyRS α-subunit in a complex with GSAd allows comparison between both types of GlyRS. To function in a meaningful biological context, these synthetases must exclude any amino acid other than glycine, including the near-cognate alanine. This recognition problem was solved in different ways by the two types of GlyRSs, which further highlight their distinct origin.

Both types of GlyRSs share 1) the same basic architecture of class II synthetases, a six-stranded antiparallel β-sheet. However, the active site domains are not similar enough to share the same subclass, 2) the same ATP-binding site, which is a common feature of all class II synthetases, built using residues of the characteristic motifs 2 and 3. However, the motif 2 signature is different in the two types of GlyRSs, 3) a glutamic acid residue (Glu-156) that helps to recognize the amino moiety of the cognate amino acid glycine.

Beyond these general similarities, there are key differences at the molecular level between the two types of GlyRSs. First, other than the class II antiparallel β-sheet, there are no other structural elements or residues shared by the two types of GlyRS in this domain. Second, the character of the eukaryotic GlyRS dimerization interface is mainly hydrophobic like most class II synthetases, whereas that of the bacterial GlyRS is stabilized by a number of salt bridges. Third, the most impressive difference between the two classes of GlyRSs is their highly distinct strategies for amino acid recognition. Whereas bacterial GlyRS interacts with the amino group of glycine through five absolutely conserved residues, eukaryotic GlyRS uses three absolutely conserved glutamic acids, creating an electronegative pocket, to perform this task. To further illustrate the different character of this region, an absolutely conserved residue in eukaryotic GlyRS (Arg-410) that interacts with two highly conserved residues, Glu-245 and Glu-522, is substituted by a highly conserved Phe (142) in bacterial GlyRS.

The dynamics of the reaction, exemplified by the amino acid loop and its topological analog in bacterial GlyRS, also differs in both types of GlyRS. In eukaryotic GlyRS, the hydroxyl group of a non-conserved Tyr residue contacts the carboxyl moiety of the cognate glycine. In bacterial GlyRS, an absolutely conserved Trp (115) residue closes the amino acid binding cavity, forming a cation-π interaction with the amino group of the cognate glycine. Finally, the discrimination of the near-cognate alanine is made in bacterial GlyRS by two nearly absolutely conserved Thr residues (140 and 158) that also bind a solvent molecule, although in eukaryotic GlyRS this task is done mainly through a non-conserved Ser or Ala residue (524), equivalent of Thr-158 (Fig. 4*B*). The equivalent of Thr-140 is a non-conserved position (Ala, His, or Ser-408) in eukaryotic GlyRS.

Such disparate differences have only been partially observed until now in the case of SerRSs, where a subset of methanogenic archaea possesses a SerRS with different amino acid recognition elements than those found in the majority of SerRSs (48). However, in this case, both types of SerRS appear to belong to subclass IIa of aaRSs (Figs. 8, 9*C*, and 10 and PDB entries 2dq0 and 2cj9). Overall, the features of the homologous catalytic core of the two types of GlyRSs are so different that it is extremely unlikely for them to share a common origin.

##### There Are Four Structural Subclasses of Class II aaRSs

We used the standard accepted structure and sequence comparison methods to detect structural relationships, including the following: an all-against-all similarity matrix using Dali Z-scores (41, 49, 50); a dendrogram built according to the overall Cα r.m.s.d. values derived from the STAMP superposition algorithm (42) as implemented in MultiSeq (51, 52); a PCA and r.m.s.d. dendrogram based on a conserved core of 80 Cα atoms using bio3D (44); and a dendrogram built with PhyML (53) according to a weighted multiple sequence alignment made using structural information, as implemented in T-Coffee Expresso (43) and validated using Transitive Consistency Score (TCS) (54). Using these tools and the crystal structures from several species and in complex with different ligands, we were able to define four different class II divisions that are in general agreement with previous proposals. We made this classification proposal based mainly on the structural features of a monomeric catalytic core. Remarkably, even if this core is reduced to a minimal structure of 80 Cα atoms, it is still possible to define the four subclasses. It is noteworthy that the new adjustment of class IIc and IId takes into account the physicochemical character of the cognate amino acids, the aminoacylation site, and correct quaternary structures among the four proposed subclasses (Table 3). PheRS and SepRS are clear functional outsiders from the subclass IId (AlaRS and bacterial GlyRS); the corresponding amino acids are much bigger than Ala and Gly; the aminoacylation sites are different (3′-OH for AlaRS and GlyRS and 2′-OH for Phe and SepRS (27)), and the quaternary structures are also different, most notably between bacterial GlyRS (α_2_β_2_) and PheRS (αβ)_2_ (Table 3). In this way, subclasses IIc and IId encompass much more homogeneous and coherent members, as opposed to the previous subclass IIc, which included a wide variety of aaRSs (25–27).

These analyses define a clear subgroup for AlaRS and bacterial GlyRS. According to the sequence analysis, however, it is less clear how to classify PylRS, which has been suggested to belong to subclass IIc (55) (consistent with our structure-based classification) or IIb (56) (as in our sequence analysis). There also appears to be a discrepancy in the case of HisRS at the sequence level. However, HisRS can be confidently placed in class IIa due to the presence of an anticodon recognition domain located on the C-terminal region, which is another signature of this subclass.

Recent phylogenetic trees have not commented on the proposed subdivision (27, 55). However, a relationship between AlaRS and bacterial GlyRS has even been suggested based on the insertions in the catalytic core and the C-terminal helical domain (57, 58). To address this, in this work we presented a complete analysis of all class II synthetases, defined a new subclass, and explored the similarities between AlaRS and bacterial GlyRS.

##### Bacterial GlyRS and AlaRS Are Highly Related

In contrast to its comparison with eukaryal GlyRS, bacterial GlyRS shares several key features with AlaRS. First, these two enzymes display similar catalytic cores whose architectures are distinct from other subclasses. Second, they present a similarly modified motif 2 signature: an insertion between an Arg and an acidic residue and a variation in this position of Glu for Asp, where the overwhelming consensus is a Glu residue in most class II aaRSs. Third, in the active site, they share the same strategy for the recognition of the amino group. Fourth, they share an absolutely conserved Trp involved in the recognition of the cognate amino acids and a highly conserved Ala in the vicinity of this site. An interesting case of a structural/functional convergence is seen in this particular feature in methanogenic archaea SerRS, where a different motif approaches a Trp (396) in a different conformation but with the same apparent role (PDB code 2cj9). Fifth, outside of the active site, AlaRS and bacterial GlyRS share four other highly conserved residues.

##### Proposed Models for the Origin of the Two Types of GlyRS

GlyRSs exhibit an unusual scenario for two enzymes with the same activity and the same overall fold of the catalytic domain. In most cases, these similarities would indicate that they share a common origin. Accordingly, we may consider different scenarios for the structural source of the catalytic core of GlyRSs as follows. 1) A single common ancestor, where an initial α_2_β_2_ GlyRS gave rise to another GlyRS α_2_ or vice versa. We consider this hypothesis unlikely, according to the analysis presented in this work. 2) Two different ancestors with an emergence of a dimeric eukaryotic type α_2_ from an ancient ancestor for all class II synthetases and another emergence of α_2_β_2_ GlyRS from AlaRS or a pre-AlaRS.

We believe there are several points that support this second hypothesis. First, there is high structural and sequence similarity between the catalytic cores of AlaRS and bacterial GlyRS. Second, the amino acids Ala and Gly are related in nature, only differing by one methyl group and thus may be recognized in a similar manner. In fact, it is possible to delineate changes in bacterial GlyRS in the positions of Thr-158, Thr-140, and/or Glu-256 or in the equivalent and absolutely conserved Gly-250, Val-215, and Asp-248 positions of AlaRS to partially interconvert the amino acid specificity (45, 59). Third, AlaRS presents editing activity against Gly and Ser to cope with the misincorporation of these amino acids (1/300 for Ser and 1/170 for Gly) in the active site (60). It is possible that a pre-AlaRS, able to aminoacylate both Ala and Gly, may have given rise to GlyRS. Taken together, we favor that the second scenario would more easily solve the paradox of the presence of two proteins with the same function and sharing the same primordial fold but not a single common ancestor. The two types of GlyRS represent a beautiful case of isofunctional paralogs between species (61).

## Experimental Procedures

### Protein Purification

The cDNA coding for α-AaGlyRS was synthesized by Genscript, optimized according to *E. coli* codon frequency, and subcloned into the pET-28 vector (Novagen). Recombinant α-AaGlyRS was expressed with an N-terminal His_6_ affinity purification tag in *E.coli* BL21(DE3) cells. Cells were grown at 37 °C until an *A*_600_ of 1.0 was reached. At that time, cells were induced for 10 h with a final concentration of 1 mm isopropyl β-d-thiogalactopyranoside at 37 °C and were harvested by centrifugation (5515 × *g* for 20 min). The resulting cell pellet from 2 liters of culture was frozen at −80 °C until needed. For α-AaGlyRS purification, the cell pellet was thawed and then resuspended in 20 ml of buffer A (50 mm sodium phosphate, pH 6.3, 100 mm potassium chloride, 1 mm MgCl_2_). Cells were lysed by sonication on ice and then centrifuged at 20,410 × *g* for 30 min. The resulting supernatant was heated at 75 °C for 30 min and then centrifuged again at 20,410 × *g* for 30 min. The supernatant was supplemented with the following additives: 1 m KCl, 0.5 m urea, 50 mm imidazole, and 10% glycerol (final concentrations) and applied to a 5-ml Ni^2+^-Sepharose FF column (GE Healthcare) connected to an Äkta FPLC system (GE Healthcare). The column was then washed with buffer A plus additives to remove nonspecifically bound proteins. The target protein was eluted with a 100-ml gradient of buffer A plus 500 mm imidazole. Fractions containing α-AaGlyRS were pooled and concentrated (Amicon Ultra-filter Millipore, 30 kDa) to 15 mg/ml. The protein was >99% pure as judged by denaturing gel electrophoresis. The protein was loaded onto a size exclusion chromatography Superdex S-75 10/300 analytical column (GE Healthcare) connected to DAWN Heleos-II and Optilab RI detectors (Wyatt Technologies). The column was run at a flow rate of 0.5 ml/min. One peak was eluted that included 99.9% of the injected mass and corresponded to the dimeric form of α-AaGlyRS, according to the light scattering measurements. Prior to crystallization, the protein was desalted into 20 mm HEPES, pH 7.2, 50 mm NaCl, and 1 mm DTT.

### Crystallization and Structure Determination

Crystals of α-AaGlyRS were obtained by sitting-drop vapor diffusion. Drops containing 1 μl of α-AaGlyRS at 15 mg/ml yielded crystals at 18 °C when mixed with 1 μl of a reservoir solution containing 30% polyethylene glycol monomethyl ether 2000 and 100 mm potassium thiocyanate. Crystals were soaked for 2 days in a solution containing a final concentration of 28 mm GSAd by mixing 1.3 μl of the drop containing crystals, 2.5 μl of reservoir solution, and 1.5 μl of GSAd at 100 mm. The synthesis of GSAd was performed as described previously (62). Crystals were cryoprotected in a solution prepared with the mother liquor supplemented with 15% glycerol and then flash-frozen in liquid nitrogen. Diffraction data were collected at 100 K using a wavelength of 0.9785 Å at the Life Sciences Collaborative Access Team (LS-CAT) 21-ID-G beamline at the Advanced Photon Source (Argonne National Laboratory, Argonne, IL). Data were indexed and processed with iMOSFLM (63) and reduced with Aimless (64). The structure was solved by molecular replacement using Balbes (65), with a final search model based on the structure of the α-subunit GlyRS from *Thermotoga maritima* (PDB code 1j5w). There are five monomers in the asymmetric unit, with a crystal solvent content of 57%. The initial *R*_free_ was 0.35. The ligand GSAd was fitted by means of the LigandFit program of the PHENIX suite (66). Refinement was alternated with manual building/refinement in COOT (67), PHENIX, and the PDB_REDO server (68). Non-crystallographic symmetry restraints were used for the refinement. The model presents no Ramachandran outliers, with 1355 residues (97%) on the favored and 36 residues (3%) on the allowed regions. *B*-factor refinement was limited to one value per amino acid. Monomers A and B present the best adjustment to the electron density according to the wwPDB validation report (0 residues with RSR-Z scores >2), and all figures were prepared using these monomers as templates. Two solvent molecules were located near the glycine recognition site. They could be well fitted and seen on double difference and omit maps for monomer B, and they can be seen as peaks bigger than 3.5σ on difference, *F_o_* − *F_c_* electron density maps on the four other monomers. No other solvent molecules were added to the model. σA-weighted 2*F_o_* − *F_c_*-simulated annealing omit maps were used to validate further the quality of the model. Data collection and refinement statistics are summarized in Table 1. Analysis of the interface was performed with the PISA server (69). Because of the absence of ideal geometric parameters from MSDchem, the geometry of the GSAd ligand was adjusted to that observed in PDB code 3hy0 (structure of catalytic fragment of *E.coli* AlaRS in the complex with GlySA). Validation of the GSAd ligand was performed with the ValLigURL server (70). Figures were prepared with PyMOL (The PyMOL Molecular Graphics System, Version 1.7.4, Schrödinger, LLC).

### Thermal Shift Assays

According to the protocol of Ref. 71, purified α-AaGlyRS in 25 mm NaH_2_PO_4_, 25 mm KCl, and 10 mm MgCl_2_, at a final concentration of 1 mg/ml, was mixed with different concentrations of ligands and a 1:100 dilution of SYPRO Orange dye (Invitrogen) in a final volume of 10 μl. The dye was excited at 490 nm and the emitted light intensity was recorded at 575 nm. Data were collected at 1 °C intervals from 25 to 99 °C on a StepOnePlus real time PCR system and analyzed using the Protein Thermal Shift software from Applied Biosystems. No significant differences were detected using three different values of pH (5, 7, and 8). Assays were performed in triplicate.

### Measurements of SAXS Data

Data collection for SAXS of α-AaGlyRS was performed following standard procedures in the European Molecular Biology Laboratory (EMBL) on the storage ring Petra III (DESY, Hamburg, Germany) on the P12 beamline (72). The scattered intensity was recorded as a function of the scattering vector *s* using a wavelength of 0.124 nm at 23 °C. The measurements were carried out in 30 mm HEPES, pH 7.4, 25 mm NaCl, and 1 mm DTT, with exposure times of 20 × 0.05 s. The average of the data was normalized and subtracted the scattering attributed to the solvent using automatic procedures (73). The SAXS data were processed using PRIMUS (73), where the values of the forward scattering intensity *I*_0_ and radii of gyration *R_g_* were evaluated from the experimental SAXS patterns using the Guinier approximation; these parameters were also computed from the entire scattering curve using Porod's law by the calculation of the distance distribution function *P*(*r*) using the program GNOM (74). Three different approaches were used to determine the molecular weight as follows: the *I*_0_ value, the Porod volume (from the *Pr* function), and the excluded volume (from DAMMIF calculations). The low resolution shape was reconstructed *ab initio* by the DAMMIF method (75). The theoretical SAXS patterns of dimer and monomer from the crystallographic structure were predicted using CRYSOL (76) and were compared graphically with the experimental data using Sasplot (73). The missing flexible residues of the His tag/tobacco etch virus were modeled using Coral (77). The crystallographic structure with the flexible residues was superposed with the *ab initio* envelope using Supcomb (78).

### Activity Assays

#### 

##### Glycine Activation

The first step of the aminoacylation reaction was measured using a previously described method to quantify the synthesis of aminoacyl-adenylate (79). Reaction conditions were initially screened for optimization of pH, enzyme, ATP, and glycine concentrations. The final reaction mix contained the α-subunit of Aa-GlyRS at 40 μm in 50 mm Tris, pH 8.0, 50 mm KCl, 10 mm MgCl_2_, 0.5 mm ATP, 0.25 μCi of [α-^32^P]ATP and varying concentrations of glycine. Reactions mixes were pre-incubated for 5 min at 45 °C and started by the addition of enzyme. 1-μl time points were quenched in 4 μl of buffer containing 0.1% SDS and 400 mm sodium acetate, pH 5.2. 2-μl aliquots were spotted on pre-washed PEI-cellulose thin layer chromatography (TLC) plates and developed in 0.1 m ammonium acetate, 5% acetic acid solution. The TLC plates were dried and exposed to a phosphor-screen overnight to monitor Gly-[^32^P]AMP, [^32^P]AMP, and [^32^P]ATP.

Images were obtained on an Amersham Biosciences Storm 820 Imager and quantified with ImageQuant Tl version 2005. The total fraction of [^32^P]AMP was plotted against time to obtain initial velocities; the slopes from the linear data were then plotted against glycine concentration and fit to a hyperbolic Michaelis-Menten equation using Prism6 software. All experiments were performed in triplicate.

##### Aminoacylation Reactions

According to Refs. 80–82, the aminoacylation of *A. aeolicus* tRNA^Gly^ was assayed using identical buffer conditions as for the glycine activation reaction, with the exception of using 5 mm ATP and the omission of labeled ATP. Instead *in vitro* transcribed tRNA^Gly^ was radiolabeled at the 3′-end using [α-^32^P]ATP and the *E.coli* tRNA nucleotidyltransferase, and labeled tRNA was purified using a QIAquick nucleotide removal kit (Qiagen), followed by buffer exchange using Amicon Ultra 3-kDa filter (Millipore). All reactions were initiated by the addition of enzyme, and 1-μl aliquots were quenched in 10 μl of buffer containing 0.1% SDS and 400 mm sodium acetate, pH 5.2, and 0.1 mm P1 nuclease SIGMA (N8630-1VL) or 0.1% SDS, 400 mm sodium acetate, pH 5.2. 0.5 μl of the quenched mix were spotted on pre-washed PEI-cellulose TLC plates and developed and treated like the ones for the glycine activation reaction.

### Structural and Sequence Analysis

#### 

##### STAMP

A collection of 111 crystal structures from class II aaRSs was chosen according to their diversity in specificity, species, and ligands bound in the active site to take into account conformational changes. The catalytic cores of these structures was selected by including the residues located between motifs 1 and 3 and excluding additional insertions or domains not comprising common structural motifs for most aaRSs. An example of the trimmed catalytic cores is found in Fig. 6. The PDB entries of the structures used and a partial description of them are found in supplemental Fig. S1. The approximate length of each structure of the working set was 200 residues. An overall multiple structural alignment was made using the STAMP algorithm (42) as implemented in MultiSeq in VMD (51, 52). The program generates trees according to four different criteria as follows: Qres (structure similarity per residue); Qh (structural homology); percent identity, and r.m.s.d. Bacterial GlyRS and AlaRS appeared in a separate group in the trees calculated according to all four parameters. Although all the trees were highly similar to each other, the percent identity and r.m.s.d. trees were in full agreement with previous subclass definitions. The same results were obtained when the algorithm was applied to just a sample of representative aaRSs.

Structure-based multiple sequence alignments were made for the cases of the two types of GlyRSs and AlaRS. All the sequences contained in Pfam families PF01411 (AlaRS), PF02091 (bacterial GlyRS) and PF00587 (subclass IIa, but trimmed for eukaryal GlyRS) were analyzed. The catalytic cores were selected and aligned with the ClustalW plug-in in MultiSeq. Non-redundant sets were generated using the sequence QR tool in MultiSeq (83). A profile alignment was calculated taking into account the structures aligned for each type of aaRS.

##### DALI

A sample of structural cores as defined above was used to perform an all-against-all three-dimensional structural comparison with the Dali pairwise comparison server. A matrix was generated based on the resulting Z-score values. Although no tree was derived from this matrix, four clear groups are defined, taking into account a cutoff of 18.5. The Z-score value for structures belonging to other subgroups was between 11.9 and 16.5. In most cases other than PylRS, a clear gap is seen between members and non-members of the subclass.

##### T-Coffee Expresso

A non-redundant set of sequences of the catalytic core of 63 structures from different species used for the alignment with STAMP were aligned using structural information with the T-Coffee Expresso server (43). The obtained alignment was further validated by means of the suggested TCS method, which identifies the most correct positions on the alignment (54). An estimated phylogenetic tree was built with PhyML (53) according to the tcs_weighted obtained file, where the sequences are ordered according to their TCS score. The tree was visualized with Archaeopteryx (84).

##### Root Mean Square Deviation and PCA with Bio3D

The analysis was performed using the Bio3D package in R (44). 39 structures derived from the STAMP analysis were selected, three of different species and in complex with different ligands for each type of aaRS. The aligned structures were used in Bio3D to define an invariant core with the minimal structural variance among all the protein structures. This core of 80 Cα atoms was the basis for the r.m.s.d. dendrogram calculation and PCA. The structures were superimposed onto this core, and the variances shown by PC1 and PC2 were used to define the four different subgroups.

## Author Contributions

M. I. V. S., A. T. L., A. C. D. B., and D. M. conceived the project. M. I. V. S. and H. A. S. S. purified α-AaGlyRS and performed thermal shift assays. A. R. H. and L. B. C. performed the activity experiments. M. I. V. S. purified, crystallized, and solved the structure of the α-AaGlyRS-GlyAMS complex. M. I. V. S., H. M., and D. S. performed and analyzed the SAXS experiments. R. F. and M. G. synthesized the Gly-SA compound. D. M., B. B., M. A., M. I. V. S., and A. T. L. performed the structural and sequence analysis. All authors analyzed the data and contributed to manuscript preparation. M. I. V. S. and A. T. L. wrote the manuscript.

## Supplementary Material

Supplemental Data

## Footnotes

4 The abbreviations used are:

aaRS: aminoacyl tRNA synthetase
Gly-SA: G5A, or GSAd, 5′-*O*-[*N*-(l-glycyl)sulfamoyl] adenosine
SAXS: small angle x-ray scattering
TCS: transitive consistency score
SEC-MALS: size exclusion chromatography coupled to multiangle light scattering
PCA: principal component analysis
r.m.s.d.: root mean square deviation
PDB: Protein Data Bank
GlyRS: glycyl tRNA synthetase
HisRS: histidyl tRNA synthetase
ThrRS: threonyl tRNA synthetase
ProRS: prolyl tRNA synthetase
SerRS: seryl tRNA synthetase
PheRS: phenylalanyl tRNA synthetase
AlaRS: alanyl tRNA synthetase
SepRS: phosphoseryl tRNA synthetase
PylRS: pyrrolysyl tRNA synthetase.
