# Supplementary material for: Structural Insights into the Polyphyletic Origins of Glycyl tRNA Synthetases
Source: J Biol Chem. 2016 May 23;291(28):14430–46. doi: 10.1074/jbc.M116.730382 (PMC4938167; doi:10.1074/jbc.M116.730382)
Supplement: Supplemental Data [file supp_291_28_14430__index.html]

Structural Insights into the Polyphyletic Origins of Glycyl tRNA Synthetases — Structural Insights into the Polyphyletic Origins of Glycyl tRNA Synthetases — Insights into the Polyphyletic Origins of GlyRSs — Supplemental Data 

# Structural Insights into the Polyphyletic Origins of Glycyl tRNA Synthetases

## Supplemental Data

- Supplemental data (.doc, 1.1 MB) - Supplemental data (dendrograms)
