# Supplementary material for: Structural Insights into the Polyphyletic Origins of Glycyl tRNA Synthetases
Source: J Biol Chem. 2016 May 23;291(28):14430–46. doi: 10.1074/jbc.M116.730382 (PMC4938167; doi:10.1074/jbc.M116.730382)
Supplement: Supplemental Data [file 10.1074_M116.730382_jbc.M116.730382-1.doc]

Supplementary material

Structural Insights into the Polyphyletic Origins of Glycyl tRNA Synthetases

**Marco Igor Valencia-Sánchez^1^, Annia Rodríguez-Hernández^1,2^, Ruben Ferreira^3^, Hugo Aníbal Santamaría-Suárez^1^, Marcelino Arciniega ^1^, Anne-Catherine Dock-Bregeon^4^, Dino Moras^5^, Brice Beinsteiner^5^, Haydyn Mertens^6^, Dmitri Svergun^6^, Luis Brieba de Castro^2^, Morten Grøtli^3^, and Alfredo Torres-Larios^1^***


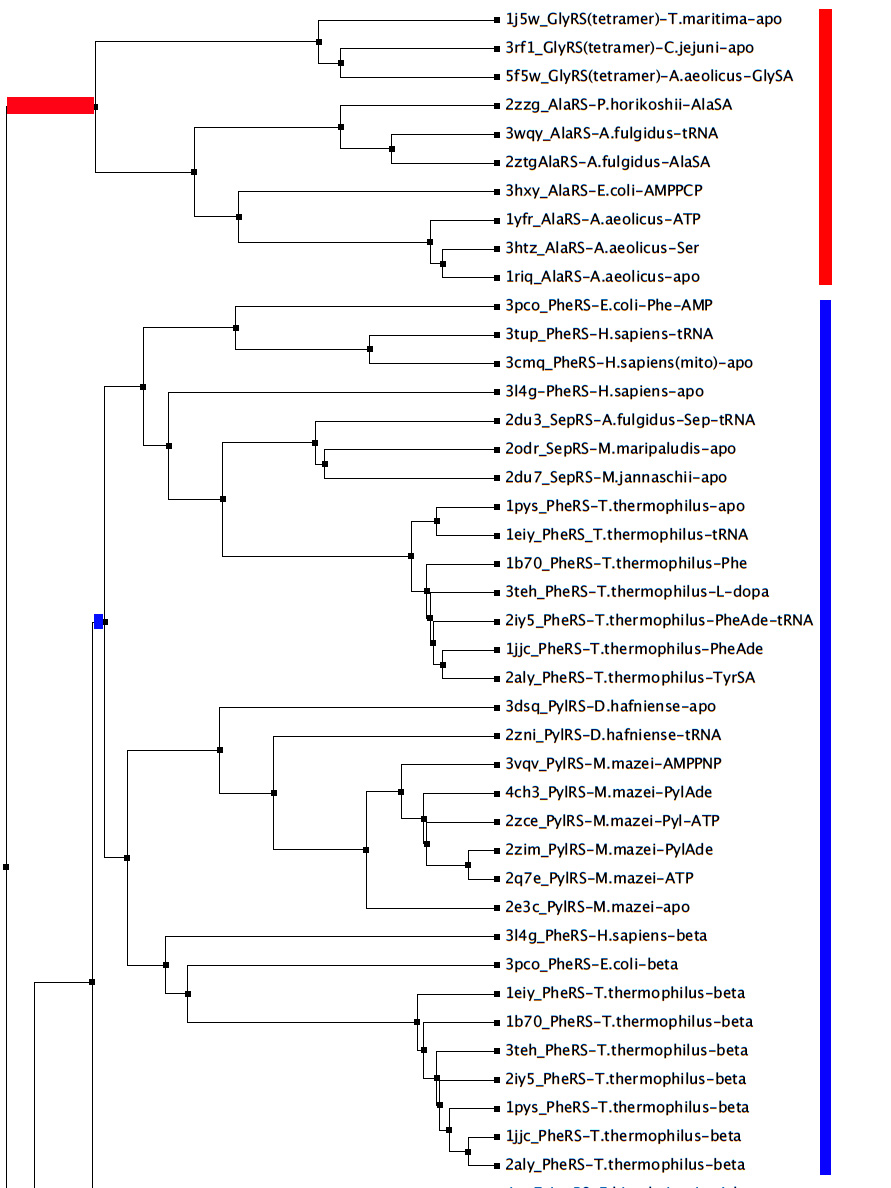


**Supplementary Figure 1. Continued on next page.**


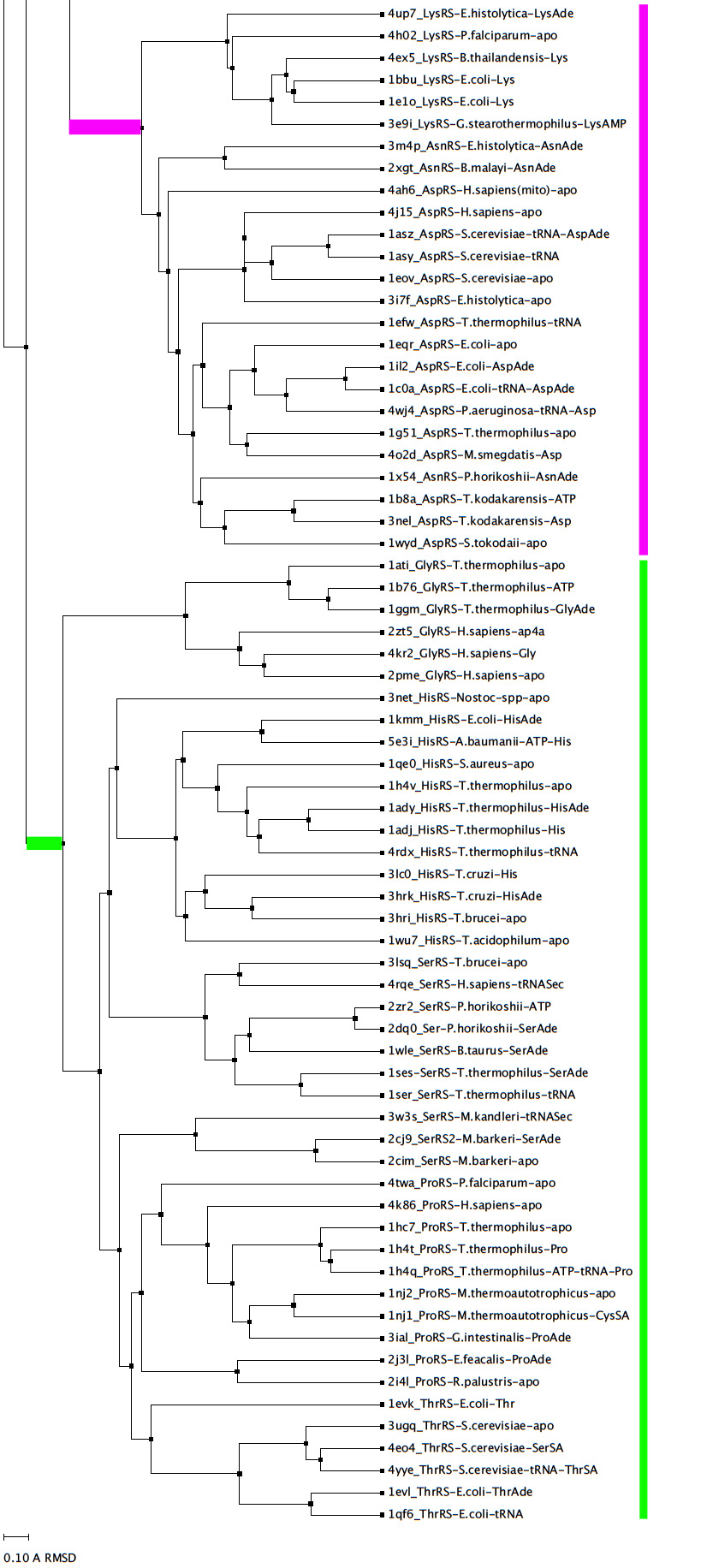


**Supplementary Figure 1. Dendrogram according to the RMSD values obtained from STAMP as implemented in Multiseq.**


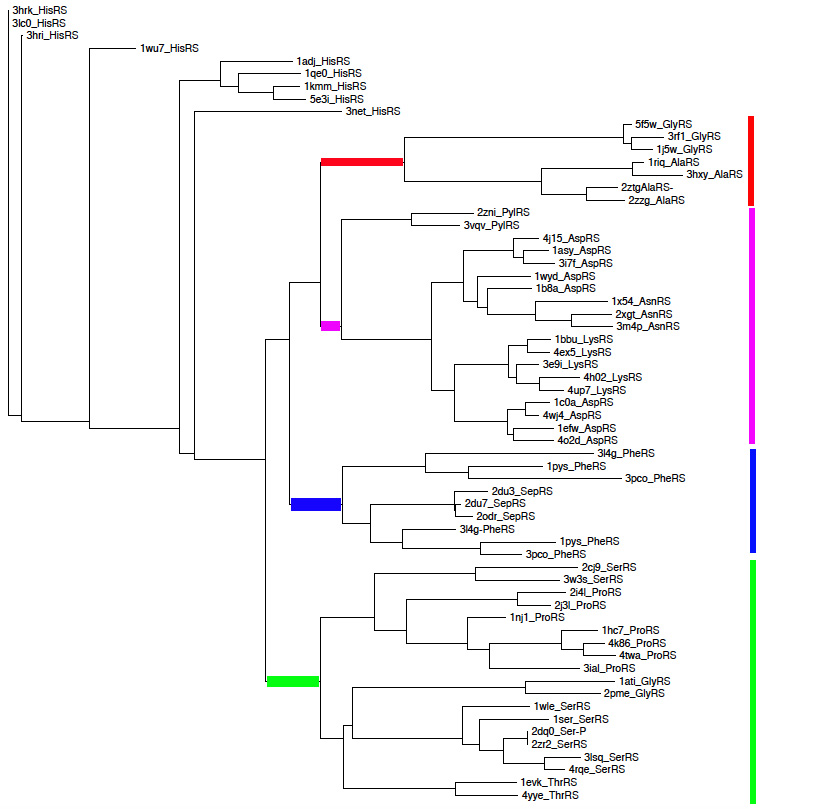


**Supplementary Figure 2. Dendrogram according to sequence features.** The tree was obtained by means of the use of T-Coffee Expresso, transitive consistency score and PhyML.
